# Responses of Five Different Artificial Intelligence Chatbots to the Top Searched Queries About Erectile Dysfunction: A Comparative Analysis

**DOI:** 10.1007/s10916-024-02056-0

**Published:** 2024-04-03

**Authors:** Mehmet Fatih Şahin, Hüseyin Ateş, Anıl Keleş, Rıdvan Özcan, Çağrı Doğan, Murat Akgül, Cenk Murat Yazıcı

**Affiliations:** 1 Faculty of Medicine Department of Urology, Tekirdağ Namık Kemal University, Süleymanpaşa, Tekirdağ, 59020 Turkey; 2 Department of Urology, Bursa State Hospital, Nilüfer, Bursa, 16110 Turkey

**Keywords:** Artificial intelligence, Bard, Bing, Chatbot, ChatGPT, Copilot, Erectile dysfunction, Ernie bot

## Abstract

The aim of the study is to evaluate and compare the quality and readability of responses generated by five different artificial intelligence (AI) chatbots—ChatGPT, Bard, Bing, Ernie, and Copilot—to the top searched queries of erectile dysfunction (ED). Google Trends was used to identify ED-related relevant phrases. Each AI chatbot received a specific sequence of 25 frequently searched terms as input. Responses were evaluated using DISCERN, Ensuring Quality Information for Patients (EQIP), and Flesch-Kincaid Grade Level (FKGL) and Reading Ease (FKRE) metrics. The top three most frequently searched phrases were “erectile dysfunction cause”, “how to erectile dysfunction,” and “erectile dysfunction treatment.” Zimbabwe, Zambia, and Ghana exhibited the highest level of interest in ED. None of the AI chatbots achieved the necessary degree of readability. However, Bard exhibited significantly higher FKRE and FKGL ratings (*p* = 0.001), and Copilot achieved better EQIP and DISCERN ratings than the other chatbots (*p* = 0.001). Bard exhibited the simplest linguistic framework and posed the least challenge in terms of readability and comprehension, and Copilot’s text quality on ED was superior to the other chatbots. As new chatbots are introduced, their understandability and text quality increase, providing better guidance to patients.

## Introduction

Erectile dysfunction (ED) is a prevalent urologic disorder worldwide, resulting in more than 2.9 million outpatient visits annually in the United States alone [1]. Despite its prevalence, ED is often neglected or improperly managed, and men experience significant barriers in openly addressing the condition with their doctors. Reportedly, only 32.4% of men feel comfortable initiating a conversation about ED, and only a mere 10.5% can confidently state that their doctor has raised the topic [2]. Although sexual education is widely accessible, sexual health issues remain a difficult subject to talk about and a significant obstacle in oppressive countries [3]. Digital advancements such as chatbots can provide patients with access to information and therapy without the need for direct human involvement; hence, they have the potential to significantly impact the diagnosis and treatment of ED [4]. However, online health information lacks substantial regulation, resulting in a highly variable quality of information.

Artificial intelligence (AI) chatbots are software programs that act as virtual assistants and provide services to users via natural language interactions on social media platforms or web-based apps [5]. Studies revealed a rising use of AI chatbots, which are transforming the way people engage with technology by embracing a more sociable and conversational method, leading to enhanced user experience [6]. AI chatbots can be used in several domains of healthcare such as customer support and symptom detection to help users assess the need to contact a healthcare expert. Patients with andrological diseases, such as ED, need reliable and accurate health information that is both generic and specialized for their treatment. Due to embarrassment, patients with ED turn to AI chatbots to seek solutions for their condition. AI chatbots can assist these patients by monitoring their status, providing personalized information, and encouraging medication adherence [7]. Nevertheless, there are obstacles, and apprehensions, in obtaining health-related information online due to their lack of precision and dependability. In addition, those with limited proficiency in medical language may have difficulties in evaluating the trustworthiness and validity of the information obtained.

While previous studies have evaluated ED content on ChatGPT, studies comparing the readability and quality of responses produced by various chatbots on the same topic are scarce [8]. This study aimed to evaluate and compare the quality and readability of information generated by five different AI chatbots on the most popular keywords of ED.

## Materials and Method

This study was conducted on January 20, 2024, at the Urology Department of Tekirdag Namik Kemal University. As the study did not include any procedures on living organisms or human data, obtaining ethical committee approval was not required. Before conducting the searches, all personal browser data were erased as a precautionary measure to avoid bias. Google Trends (https://trends.google.com/) was used to ascertain the frequently searched ED-related phrases [9]. The search queries were collected from global searches conducted between 2004 and January 20, 2024. A comprehensive list of the top 25 most often searched phrases was compiled, including a diverse array of subjects in Google’s online search queries. Five terms were omitted from the study due to their irrelevance to the issue or their brevity and lack of completeness: “Ed,” “Viagra,” “Testosterone,” “Prostate,” and “Diabetes.” Subregions were used to classify and record the geographical areas of significance.

The provided search terms were methodically entered into ChatGPT January 24 Version (https://chat.openai.com/), Bard Version 2.0.0 (https://bard.google.com/), Bing Chat (https://www.bing.com/chat), Copilot (https://copilot.microsoft.com/), and Ernie Bot 4.0 (https://yiyan.baidu.com/) while preserving the precise sequence of the original searches. As mentioned previously, all browser data was completely erased before starting the searches, and separate accounts were created for interacting with each AI chatbot to guarantee a significant distinction. Every inquiry was processed on a separate chat page to ensure separation and optimize the analytic procedure. The resulting responses were stored for further assessments of readability and quality.

The assessment of the quality of the acquired texts was performed using the Ensuring Quality Information for Patients (EQIP) tool, which evaluates the different aspects of content, such as the coherence and quality of writing. The questionnaire consists of 20 inquiries, with response options including “yes,” “partly,” “no,” or “does not apply.” The scoring approach entails the multiplication of the quantity of “yes” responses by 1, “partly” responses by 0.5, and “no” responses by zero. The resultant values are aggregated, divided by the total quantity of items [20], and adjusted by removing the count of responses labeled as “does not apply.” The EQIP score, expressed as a percentage, is obtained by multiplying the final value by 100. The final averaged EQIP score was used to classify each resource. The classification criteria were determined using score ranges that were as per the guidelines specified in the original EQIP development paper [10]. Resources with scores ranging from 76%–100% were categorized as “well written,” indicating exceptional quality; 51%–75% as “good quality with minor issues”; 26%–50% as “serious quality issues”; and 0%–25% as “severe quality issues” [11].

The evaluation of the accuracy of the data in each passage was conducted using the DISCERN questionnaire, a validated instrument developed to assist both information providers and patients in assessing the quality of written content on treatment possibilities. In addition, the questionnaire aims to promote the creation of reliable and scientifically supported health information for consumers by establishing criteria and acting as a guide for writers. The instrument consists of 15 questions and allows for assessment on a scale ranging from 1–5 [12]. For EQIP and DISCERN, M.F.Ş., H.A., and A.K. conducted the evaluation procedures; ÇD was consulted in situations where inconsistencies arose. Kappa statistics were employed to assess the degree of inter-rater reliability.

The readability of the information produced by the AI chatbots was assessed using the Flesch-Kincaid Grade Level (FKGL) and Reading Ease (FKRE) criteria. FKGL calculation involves many steps: dividing the total word count by the total sentence count, multiplying the result by 0.39, dividing the total syllable count by the total word count, and finally multiplying the result by 11.8. The acquired values are added together, and 15.59 is subtracted from the total value to approximate understanding, taking into account parameters such as phrase length and syllable count. A lower score signifies enhanced understanding, and a higher score implies complex linguistic intricacy. Conversely, the FKRE formula measures the readability of a text by multiplying the average sentence length (total words/total sentences) by 1.015 and the average number of syllables per word (total syllables/total words) by 84.6. The resulting difference is then subtracted from 206.835. While a higher Reading Ease score signifies more readability, a lower level implies increased complexity [13].

Statistical analysis was conducted using SPSS version 25 (IBM, New York, USA). The data’s normality was assessed using the Shapiro–Wilk test. The mean value and standard deviation were used to examine continuous data, whereas frequency was used to express categorical data. The Kruskal–Wallis test was used to evaluate group differences and means. The p-value was established at 0.05, resulting in a confidence interval of 95%.

## Results

The top three keywords were “erectile dysfunction cause,” “how to erectile dysfunction,” and “erectile dysfunction treatment.” A total of five keywords were eliminated (Table 1).

**Table 1:** Google Trends data of the 25 most significant keywords queried globally for ED between 2004–2023 and their classification according to EQIP

**Rank**	**Keyword**	**Relevance**	**Classification of the Topic According to EQIP**
**1**	Erectile Dysfunction Cause	100	Condition or Illness
**2**	How to Erectile Dysfunction	78	Condition or Illness
**3**	Erectile Dysfunction Treatment	65	Test, Operation, Investigation or Procedure
**4**	Erectile Dysfunction Causes	60	Condition or Illness
**5**	Ed	56	
**6**	What is Erectile Dysfunction	52	Condition or Illness
**7**	Viagra	45	
**8**	Erectile Dysfunction Help	40	Condition or Illness
**9**	Cure Erectile Dysfunction	35	Condition or Illness
**10**	Erectile Dysfunction Medicine	35	Test, Operation, Investigation or Procedure
**11**	Erectile Dysfunction Symptoms	32	Condition of Illness
**12**	Erectile Dysfunction Pills	31	Test, Operation, Investigation or Procedure
**13**	Erectile Dysfunction Meaning	28	Condition of Illness
**14**	Male Erectile Dysfunction	26	Miscellaneous
**15**	Testosterone	23	
**16**	Erectile Dysfunction Drugs	23	Test, Operation, Investigation or Procedure
**17**	Medicine for Erectile Dysfunction	22	Test, Operation, Investigation or Procedure
**18**	Causes of Erectile Dysfunction	21	Condition or Illness
**19**	Porn Induced Erectile Dysfunction	20	Miscellaneous
**20**	Treatment for Erectile Dysfunction	20	Test, Operation, Investigation or Procedure
**21**	Cause of Erectile Dysfunction	19	Condition or Illness
**22**	Erectile Dysfunction Medication	19	Test, Operation, Investigation or Procedure
**23**	Prostate	19	
**24**	Diabetes Erectile Dysfunction	18	Miscellaneous
**25**	Diabetes	18	

**Fig. 1:**
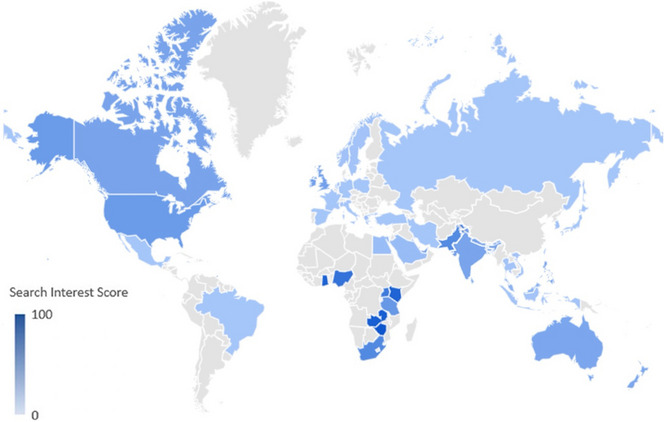
Global search interest in ED by region from 2004 to 2023 as determined by Google Trends data (regions with modest search volumes are not included)

The search interest in ED varies by country (Fig. 1). Zimbabwe, Zambia, and Ghana, with a Search Interest Score of 100, 93, and 89, respectively, ranked as the top three nations with the most search interest in ED.

**Fig. 2:**
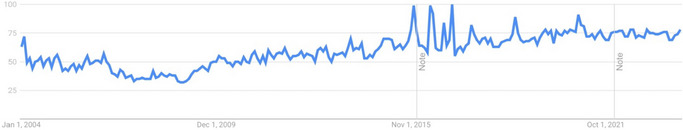
Global search interest over time, from 2004 to 2023, as determined by Google Trends data

When evaluated according to time in Google Trends analysis, it was determined that the popularity of ED has been increasing since 2009 (Fig. 2).

There was a significant difference (*p* = 0.001) in the FKRE scores among the chatbots. Applying the Bonferroni correction, a pairwise analysis revealed a significant difference in FKRE scores between ChatGPT and the other chatbots, with ChatGPT exhibiting considerably lower values and Bard with the highest score (*p* = 0.001). No other differences were noted among the remaining chatbots.

The FKGL scores between the chatbots also demonstrated significant differences (*p* = 0.001). After applying the Bonferroni correction for pairwise comparisons of FKGL scores, it was found that Bard had markedly lower FKGL scores than other bots (*p* = 0.001). No other distinctions were observed among the remaining chatbots.

The EQIP scores among the chatbots differed significantly (*p* = 0.001). Pairwise comparisons of EQIP scores using the Bonferroni correction revealed that ChatGPT had a significantly lower score compared to Ernie and Copilot (*p* = 0.001 and *p* = 0.001, respectively). In addition, there were significant differences between Bing and Ernie (*p* = 0.001), Bing and Copilot (*p* = 0.001), and Bard and Copilot (*p* = 0.001). No other differences were observed among the other chatbots.

The DISCERN scores among the chatbots also demonstrated significant differences (*p* = 0.001). After applying the Bonferroni correction for pairwise comparisons of DISCERN scores, Copilot had markedly higher DISCERN scores than the other bots (*p* = 0.001). No other differences were observed among the remaining chatbots (Table 2).

**Table 2:** Comparison of the FKRE, FKGL, EQIP, and DISCERN scores of the five different AI chatbots

	**ChatGPT**	**Bing**	**Bard**	**Ernie Bot**	**Copilot**	***p*** **value**
**Flesch Kincaid Reading Ease** ** Minimum** ** Maximum** ** Mean ± SD**	10.1 40.5 23.1 ± 7.8	25.1 61.9 39.1 ± 9.3	14.6 103 53.9 ± 21.5	25.7 55.2 37.5 ± 9.0	25.2 52.5 41.1 ± 10.1	0.001^a^
**Flesch Kincaid Grade Level** ** Minimum** ** Maximum** ** Mean ± SD**	11.6 18.2 14.3 ± 1.7	7.9 16 12.5 ± 1.8	1.9 15.2 9.6 ± 3.3	9.8 15.8 12.9 ± 1.7	10.4 15.3 12.5 ± 1.9	0.001^b^
**EQIP Score** ** Minimum** ** Maximum** ** Mean ± SD**	30.0 45.0 40 ± 4.2	35.0 45.0 39.5 ± 3.1	0 69.0 32.1 ± 30.4	0 72.2 53.1 ± 20.6	41.7 80.0 63.5 ± 12.7	0.001^c^
**DISCERN** ** Minimum** ** Maximum** ** Mean ± SD**	28.0 42.0 33.5 ± 4.8	28.0 42.0 33.3 ± 4.0	16.0 55.0 33.7 ± 16.7	16.0 44.0 32.7 ± 7.7	40.0 73.0 55.0 ± 10.6	0.001^d^

^a^Difference between ChatGPT and others

^b^Difference between Bard and others

^c^Differences between ChatGPT and Ernie, ChatGPT and Copilot, Bing and Ernie, Bing and Copilot, and Bard and Copilot

^d^Differences between Copilot and others

## Discussion

The results of this study indicated that the AI chatbots’ responses to questions concerning ED did not match the requirements for readability. While Copilot demonstrated satisfactory quality with minor flaws, Bard and Ernie Bot displayed notable quality issues Furthermore, while ChatGPT’s legibility was comparatively worse, Bard was the easiest to understand. To our knowledge, this is the first study to assess, analyze, and compare ED data obtained from different AI chatbots.

Over the years, there has been an increasing trend of interest in ED worldwide. This may be attributed to the increasing incidence of diseases causing ED. The incidence of ED is expected to increase in the future, which will lead to even greater interest in the condition. In this study, the three most frequently searched keywords were “erectile dysfunction cause,” “how to erectile dysfunction,” and “erectile dysfunction treatment.” Many people searched for the causes of ED, and finding the safest and most effective treatment options was the top priority for many men.

Africa showed the highest search interest for ED. Zimbabwe, Zambia, and Ghana ranked as the top three nations with the highest search interest for ED. This suggests that many people from these countries are actively seeking information, including potential solutions, for ED and that there is a need for awareness, education, and accessible treatments for the condition in these nations. Furthermore, Africa is expected to witness the most significant percentage of ED growth, with a predicted rise of 169% between 1995 and 2025 [14]. In a study conducted in Zimbabwe, the prevalence of ED in patients with diabetes was 73.9% [15]. In Zambia, this rate was 56%–68% [16]. Therefore, healthcare professionals in these countries should be knowledgeable regarding the prevalence and risk factors of ED and its treatment options [17].

The quality of health information is pivotal in augmenting the efficacy, cost-effectiveness, and security of healthcare provision. It also enhances patient involvement and contentment [18]. The present study revealed that while ChatGPT, Bing Chat, and Copilot demonstrated acceptable quality with minor flaws, Ernie Bot and Bard exhibited substantial quality issues. Contrary to these findings, Cocci et al. [19] observed that ChatGPT produced low-quality information on urology patients. However, the continuous improvements in AI chatbot systems are certainly accountable for the enhanced quality observed in this study [20]. Nevertheless, it is important to exercise caution when relying on health-related information obtained from Ernie Bot as Copilot has emerged as a vital source for obtaining such information. This study also emphasizes the importance of improving the material produced by AI chatbots. To achieve this, many processes could be adopted, such as facilitating the availability of medical literature and research to enhance the knowledge repository of AI chatbots. This extension could potentially enhance their ability to provide more dependable information on health-related subjects. In addition, including certain parameters tailored to healthcare data during AI model training could significantly improve their capacity to provide contextually relevant and medically accurate responses.

Online health information that is difficult to understand can lead to the dissemination of false information, possibly endangering individual health [21]. The present study revealed that the AI chatbots’ data on ED surpassed the reading level recommended by the National Institute of Health, which is typically appropriate for sixth-grade students. Temel et al. [9] found that the texts produced by ChatGPT on spinal cord injury are challenging to read. In a similar vein, Momenaei et al. [22] observed that the content produced by ChatGPT-4 on surgical treatment of retinal illnesses had elevated levels of readability. According to Önder et al. [23], the information produced by ChatGPT-4 on hypothyroidism during pregnancy would need a minimum of 9 years of education. Our study revealed that ChatGPT requires a high level of education to understand. Although Bard is comparatively easier to understand, it also needs a high education level. These results emphasize the need to ensure that AI chatbots provide precise and readily comprehensible information, particularly concerning andrological health subjects such as ED. AI chatbots with human interventions have the capacity to enhance their own readability levels. Using algorithms combined with human supervision, the produced material can be restructured to conform to specified readability standards.

The popularity of accessing online health information, particularly using technologies such as AI chatbots, is increasing. However, we maintain that in its present form, it is insufficient to substitute the need for a comprehensive medical assessment and consultation with a healthcare professional. Although internet sources can give valuable insights, they lack the individualized and comprehensive evaluation necessary for accurate diagnosis and treatment [24]. Maintaining confidentiality between a doctor and a patient with sexual health issues such as ED is important, and forming this bond is crucial for tailored therapy, which takes into account distinctive aspects that cannot be completely captured by digital contacts alone. In addition, it is essential to consider patients’ social background and their families when providing medical advice. Therefore, although AI chatbots can provide valuable insights regarding ED, including other health subjects, they should be considered only as an additional source of information and not a replacement for expert medical guidance and treatment.

This study has certain limitations. First, the search was restricted to the first 25 terms, thereby compromising the comprehensiveness of the results. By integrating new keywords, a more complete methodology might result in more precise conclusions. Furthermore, broadening the use of non-English keywords might augment the extent of the assessment, resulting in more universally relevant conclusions. Second, this study evaluated the reactions of only five AI chatbots. Given the dynamic nature of this sector and the increasing creation of novel models, future studies including a wider range of AI chatbots that may enhance the precision of the results are warranted.

## Conclusion

Of the five chatbots, Bard has the simplest language structure and is the easiest to read and understand, and Copilot has the highest text quality on ED. With the introduction of new chatbots, their comprehensibility and textual excellence improve, thus enabling them to provide enhanced counseling to patients.

## Data Availability

No datasets were generated or analysed during the current study.

## References

[1] Miller DC, Saigal CS, Litwin MS, et al (2009). The demographic burden of urologic disease in America. Urol Clin North Am; 36:11–27. 19038632 10.1016/j.ucl.2008.08.004 PMC2614213

[2] Ab Rahman AA, Al-Sadat N, Yun Low W. (2011) Help seeking behaviour among men with erectile dysfunction in primary care setting. J Mens Health.;8: S94–6.

[3] Waling A, Fraser S, Fisher C. Young People and Sources of Sexual Health Information (ARCSHS Monograph Series No. 121). Bundoora, VIC: Australian Research Centre in Sex, Health and Society, La Trobe University 2020.

[4] Russo, G.I., Asmundo, M.G., Durukan, E. et al (2023). Quality and benefits of the erectile dysfunction information on websites, social-media, and applications. Int J Impot Res. 10.1038/s41443-023-00725-1 37369784

[5] Pérez-Soler S, Juarez-Puerta S, Guerra E, de Lara J (2021). Choosing a chatbot development tool. IEEE Software; 38:94–103.

[6] Skjuve M, Brandzaeg PB (2019). Measuring user experience in chatbots: An approach to interpersonal communication competence. Internet Science: INSCI 2018 International Workshops, St. Petersburg, Russia, October 24–26, 2018, Revised Selected Papers 5: Springer;113–120.

[7] Christopherjames JE, Saravanan M, Thiyam DB, Sahib MYB, Ganapathi MV, Milton A. (2021) Natural language processing based human assistive health conversational agent for multi-users. 2021 Second International Conference on Electronics and Sustainable Communication Systems (ICESC): IEEE;1414–1420.

[8] Pan A, Musheyev D, Loeb S, Kabarriti AE (2023). Quality of erectile dysfunction information from ChatGPT and other artificial intelligence chatbots. BJU Int. 2024 Feb;133(2):152–154. Epub Nov 24. 10.1111/bju.16209 37997563

[9] Temel MH, Erden Yakup, Bağcıer Fatih (2023). Information Quality and Readability: ChatGPT’s Responses to the Most Common Questions About Spinal Cord Injury. World Neurosurgery. 10.1016/j.wneu.2023.11.062 38000671

[10] Moult B, Franck LS, Brady H (2004). Ensuring quality information for patients: development and preliminary validation of a new instrument to improve the quality of written health care information. Health expectations; 7:165–175. 15117391 10.1111/j.1369-7625.2004.00273.x PMC5060233

[11] Hain T (2002). Improving the quality of health information: the contribution of C‐H‐i‐Q. Health Expectations; 5. 10.1046/j.1369-6513.2002.00189.x PMC5060154 12199665

[12] Charnock D, Shepperd S, Needham G, et al (1999). DISCERN: an instrument for judging the quality of written consumer health information on treatment choices. J Epidemiol Community Health; 53:105–11. 10396471 10.1136/jech.53.2.105 PMC1756830

[13] Brewer J (2018). Measuring Text Readability Using Reading Level: 1499–1507.

[14] Ayta IA, McKinlay JB, Krane RJ (1999) The likely worldwide increase in erectile dysfunction between 1995 and 2025 and some possible policy consequences. BJU Int. 84 (1): 50–56. 10444124 10.1046/j.1464-410x.1999.00142.x

[15] Machingura, VPI (2018). Erectile dysfunction among diabetic patients at parirenyatwa group of hospitals in Zimbabwe. Texila International Journal of Public Health, 6(2), 69–73.

[16] Chinkoyo E, Chinkoyo E, Pather M. (2015). Erectile function in circumcised and uncircumcised men in Lusaka, Zambia: A cross-sectional study. African Journal of Primary Health Care and Family Medicine, 7(1), 1–7. 10.4102/phcfm.v7i1.766 PMC4564873 26245613

[17] Khalaf I, Levinson I (2003). Erectile dysfunction in the Africa/Middle East Region: epidemiology and experience with sildenafil citrate (Viagra^®^). Int J Impot Res 15 (Suppl 1), S1–S2. 12825101 10.1038/sj.ijir.3900967

[18] Gomes J, Romão M (2018). Information system maturity models in healthcare. Journal of medical systems;42:235. 30327955 10.1007/s10916-018-1097-0

[19] Cocci A, Pezzoli M, Lo Re M, et al (2023). Quality of information and appropriateness of ChatGPT outputs for urology patients. Prostate cancer and prostatic diseases:1–6. 10.1038/s41391-023-00754-3 37923807

[20] Howick J, Morley J, Floridi L (2021). An empathy imitation game: empathy turing test for care-and chat-bots. Minds and Machines.:1–5.

[21] Daraz L, Morrow AS, Ponce OJ, et al (2018). Readability of online health information: a meta-narrative systematic review. American Journal of Medical Quality.;33:487–492. 29345143 10.1177/1062860617751639

[22] Momenaei B, Wakabayashi T, Shahlaee A, et al (2023). Appropriateness and Readability of ChatGPT-4-Generated Responses for Surgical Treatment of Retinal Diseases. Ophthalmology Retina;7:862–868. 37277096 10.1016/j.oret.2023.05.022

[23] Onder CE, Koc G, Gokbulut P, Taskaldiran I, Kuskonmaz SM (2024). Evaluation of the reliability and readability of ChatGPT-4 responses regarding hypothyroidism during pregnancy. Scientific Reports.;14:243. 38167988 10.1038/s41598-023-50884-w PMC10761760

[24] Eysenbach G (2002). Infodemiology: the epidemiology of (mis)information. The American Journal of Medicine; 113:763–765. 12517369 10.1016/s0002-9343(02)01473-0

